# Development of a conceptual model regarding quality of life in Chinese adult patients with strabismus: a mixed method

**DOI:** 10.1186/s12955-018-0991-y

**Published:** 2018-09-03

**Authors:** Zonghua Wang, Juan Zhou, Yan Xu, Honghua Yin, Xi She, Wei Bian, Xianyuan Wang

**Affiliations:** 1 School of Nursing, Third Military Medical University (Army Medical University), Chongqing, China; 2 Southwest Eye Hospital, Third Military Medical University (Army Medical University), Chongqing, 400038 China; 3 Daping Hospital, Third Military Medical University (Army Medical University), Chongqing, China; 4 Xinqiao Hospital, Third Military Medical University (Army Medical University), Chongqing, China

**Keywords:** China, Qualitative research, Quality of life, Strabismus, Survey

## Abstract

**Background:**

Substantial challenges have been reported in China in terms of the large number of adult patients with strabismus and their poor quality of life. Quality of life is a cultural concept that varies according to personal feelings and perceptions, and it is influenced by physical, psychological and social factors. However, to date, there has been no mixed-method research of the quality of life of Chinese adult patients with strabismus, and no conceptual model has been reported. This study aimed to utilize mixed methods to explore the influence of strabismus on health-related quality of life in Chinese adult patients and to develop a conceptual model.

**Methods:**

Thirty adult patients with strabismus from three tertiary hospitals in China participated in the interview. In-depth one-to-one interviews were semi-structured and addressed strabismus-related symptoms and the impacts on the participants’ quality of life. Transcripts were analysed to identify themes. A self-designed questionnaire was distributed to 448 patients, 437 of whom returned valid questionnaires. Descriptive statistics and *x*^2^ test were conducted.

**Results:**

Five themes were revealed regarding the impact of strabismus on patient quality of life: appearance, daily activities, personal development, social interaction, and emotions. In the survey, the top three symptoms (*n* ≥ 70%) rated by the participants were monocular vision, eye fatigue and physical discomfort. Compared to those without diplopia, the patients who suffered diplopia more often reported experiencing the symptoms of blurred vision, monocular vision, physical discomfort, eye fatigue, cannot estimate depth well and increasing deviation size (all *p* < 0.05).

**Conclusions:**

This study is the first to examine quality of life among Chinese strabismus patients using both qualitative and quantitative methods and proposing a conceptual model. Symptom burden and appearance were the two original reasons for the decreased quality of life, and they were also the triggers for strabismus patients to visit clinics and undergo surgery. The interventions to treat symptoms burden should be different between patients with and without diplopia.

## Background

The concept of health-related quality of life (HRQoL) has attracted increasing popularity as an important patient-reported outcome, as it originates directly from individuals regarding their perceptions and feelings about health status [1]. The report of quality of life (QoL) generally helps health professionals and policy makers understand and evaluate whether a decision of treatment is cost-effective, as well as satisfactory to patients. QoL varies widely between people with the same illness and within an individual over time, as it is based on personal assessments and is influenced by physical, psychological and social factors.

A number of previous studies have demonstrated the reduced HRQoL in adult patients with strabismus, using either a qualitative or quantitative research design. A survey of the negative impacts of strabismus on patients older than 15 years was first reported in 1993 [2], and it revealed patients’ difficulties with self-image, employment seeking, interpersonal relationships, school, work and sports. In particular, those difficulties encountered in childhood were found to be intensified after reaching their teenage and adult years. A series of quantitative studies has also been conducted, such as the development of strabismus-specific questionnaires [3–8], the translation and validation of the questionnaires [9–14], the cross-sectional survey of QoL in strabismus patients [15, 16] and the identification of improved QoL after corrective surgery [17–22]. However, there is a limited study narrative reported in terms of the QoL in adult patients with strabismus. We identified only one study that used in-depth interviews; Holmes et al. [23] explored how strabismus affected the everyday lives of adult patients. The interview was used to develop the Adult Strabismus Questionnaire (AS-20), therefore, the results were presented as percentages to show the most frequently occurring topics. It was also found that patients with diplopia had more complaints about appearance, eye contact and interpersonal relationships.

WHO defines QoL as “an individual’s perceptions of their position in life in the context of the culture and the value systems in which they live, and in relation to their goals, expectations, standards and concerns” [1]. Thus, cultural influence on QoL should be considered when interpreting HRQoL. From this perspective, it is reasonable to assume that Chinese adult patients with strabismus could have a different perception and understanding towards QoL due to the significant difference between Chinese and Western culture. In addition, China has a vast geographical profile, and there are 56 ethnic groups, therefore, the culture differs greatly from east to west. Generally, the eastern cities have more advanced development than the western cities, in terms of economy, health and education. The medical resources are more abundant, and patients receive better care. Studies are still lacking regarding the QoL among adult patients with strabismus in western China, although our previous research has developed and validated the Chinese version of AS-20 and surveyed the QoL among adult strabismus patients in the southwest [11, 14]. However, we still recognized that it was necessary to evaluate the QoL from a qualitative perspective because this approach was an informative method of performing an in-depth investigation into patients’ views, while considering the context of culture. Qualitative and textual components could also assist researchers in identifying and clarifying concepts that were important when learning about the broader impact of strabismus.

Therefore, this study aimed to utilize a mixed-method to explore QoL in adult patients with strabismus who are living in the southwest of China and, thus, to inform the development of a conceptual model regarding QoL in strabismus. We anticipated that the conceptual model could provide readers a visual picture that clearly delineated the various concepts that were associated with the strabismus-specific QoL and represented links between the concepts. Moreover, the conceptual model could serve as a framework for ophthalmologists from which potential health intervention targets can be selected. Third, the model was expected to guide researchers’ choice of what to measure and provide a context for how to interpret the findings. Conceptual models of the impact of strabismus on patient HRQoL have not been published to date.

## Methods

### Study design and settings

To capture a full picture regarding the quality of life in adult patients with strabismus and to develop a conceptual model, we conducted a mixed-method study. First, a qualitative study adopting one-to-one semi-structured interviews was performed. This descriptive exploratory approach aimed to explore a deep and cultural understanding of the feelings and experiences of the targeted population. Then, a self-designed questionnaire was distributed among a purposive convenience sample of adult patients with strabismus at three tertiary hospitals, which aimed to collect quantitative data to confirm the links between the themes that emerged in the in-depth interviews.

### Participants

The in-depth face-to-face interviews were performed between May and July 2014, and the survey was conducted from June 2014 to Jan 2015 among adult patients with strabismus from three tertiary hospitals in the southwest of China. Strabismus patients who met the following inclusion criteria were recruited: (1) 18 years of age and older, (2) no previous strabismus corrective surgery, (3) no history of any eye-related surgery, (4) able to express themselves well, and (5) able to provide informed consent. Patients were ineligible if they had facial or ocular abnormalities or acute eye diseases that might have interfered with the study results. For the interview, the patients were purposely selected based on their demographic characteristics and clinical features to produce a heterogeneous sample. For the survey, all patients who met the inclusion criterion were invited by ophthalmologists or nurses. General information, including demographic data about age, gender, education, and socioeconomic status, and clinical features about types of strabismus and diplopia were recorded.

### Instrument

For the survey, we adopted a self-designed questionnaire, based on the preliminary interview results. Two-rounds of the Delphi expert consensus method were used to evaluate the content validation of the questionnaire. The questionnaire included 10 items evaluating strabismus-related symptoms and 24 items regarding four aspects on the negative impacts of strabismus on QoL. The four aspects were appearance, daily activities, personal development and social interaction, respectively. A 5-point Likert-type rating score was utilized for each item. The lower the score, the greater the severity of the symptoms and impacts.

### Data collection

A participant information sheet and verbal explanation were given, and all participants signed informed consent before the interview. The interviewer did not participate in the patient treatment, and participation (or not participating) did not impact the patients’ treatments. Confidentiality was emphasized to ensure individual participants could not be identified through quotations and descriptions. A code name was used to represent each participant to protect their privacy. Ethical approval was obtained from the human ethics committee of the Amy Military Medical University. The data were collected in accordance with the Code of Ethics of the World Medical Association (i.e., the Declaration of Helsinki).

Following a literature review on QoL conceptual framework and HRQoL within strabismus patients, a semi-structure interview guide was developed specifically by the research group in consultation with an advisory group consisting of nursing experts on qualitative study, a senior ophthalmologist and head nurses specialized in strabismus. Interview questions were organized to explore participants’ experience in terms of five dimensions: (1) strabismus-related symptoms, (2) the impacts of the symptoms on participants’ studies, daily activities, career and social life, (3) strategies to cope with the impacts, (4) perceived barriers and facilitators to strabismus-related quality of life, and (5) participants’ needs to improve strabismus-related QoL.

All interviews were conducted by the first author, who had received practice training for interviewing techniques. All interviews were held in a waiting room where privacy and comfort were guaranteed. Each interview was conducted face-to-face at a time convenient for each individual participant and audio-recorded with participants’ permission. Interviews varied in duration from 30 to 60 min. At the start of each interview, the purpose of the study was explained using information statements, and written informed consent was obtained from each patient before participation. An open-ended question was first asked to inspire spontaneous brainstorming of strabismus-related experiences, to avoid the introduction of bias into the data. Meanwhile, elaboration and clarification probes were used on areas of interest to encourage participants to provide in-depth descriptions of their experiences and to explain and clarify their responses to questions (e.g., “Could you please tell me a bit more about ...” or “What did you mean by ...”). Before the end of the interview, the participants were debriefed by the interviewer and invited to take part in a post-surgery interview.

An invitation was given by ward nurses to eligible adult strabismus patients to fill out the questionnaires. After giving verbal and written instructions, the participants who agreed to take part in the survey were left alone in a waiting or treating room to complete the questionnaire.

### Data analysis

All interview recordings were transcribed verbatim. Researchers listened to the recordings and read the transcripts repeatedly to ensure accuracy and to include relevant information from field notes (e.g., pauses, laughter, and overall mood of participants). The thematic analysis of the transcripts was conducted manually by two researchers, ZW and JZ. After the first three interviews, we constructed a start code book of broad categories, using both inductive and deductive approaches. An inductive reasoning allows the categories and themes to emerge as reading of transcript proceeds, while a deductive reasoning allows a further examination of the categories and themes, in relation to existing theory. Therefore, the study categories were generated through repetitive reading of the transcripts and by referencing the categories and themes addressed in the interview guide. ZW and JZ then independently coded the data for the remaining transcripts and discussed until agreement was reached. During analysis, we kept the possibility open to add any additional categories. All coded transcripts were reviewed multiple times, and new codes were added as appropriate and others were lumped together into broader categories. Saturation of themes was reached prior to the completion of 27 interviews; however, the interview continued to ensure a diverse sample, in terms of the patient demographics and disease features.

The software of Statistical Package for the Social Sciences (SPSS, Version 21.0) was adopted to analyse the numerical data. A descriptive data of headcounts and percent was produced regarding demographic information and clinical features. The differences on the reported symptoms were compared using the *x*^2^ test between the strabismus patients with and without diplopia. All statistics were two-sided and fixed at a 5% level of significance.

## Results

Thirty patients with strabismus participated in the semi-structured interview. As shown in Table 1, the participants ranged in age from 18 to 65 years (mean age, 33.2 years). Most of the interviewees were young adults (*n* = 12) and middle-aged adults (*n* = 13). The participants predominately had a low education background of secondary school and below. Approximately one-third of the participants were unemployed. Two-fifths of the participants suffered from diplopia.

**Table 1:** Demographic data on research participants

Variable	Interview participants n (Percent, %)			Survey participants n (Percent, %)
	Male	Female	Total	
Age range (yrs)	16 (53.3)	14 (46.7)	30	
18–25	5	7	12	211 (48.3)
26–45	8	5	13	177 (40.5)
46–65	3	2	5	49 (11.2)
Education level				
Secondary school and below	10	10	20	190 (43.5)
High school	4	3	7	176 (40.3)
University and above	2	1	3	71 (16.2)
Marital status				
Unmarried	7	6	13	258 (59.0)
Married	7	5	12	179 (41.0)
Divorced	1	2	3	–
Widowed	1	1	2	–
Deviation Size				
≤ 25 Pd	8	7	15	220 (50.3)
> 25 Pd	8	7	15	217 (49.7)
Diagnosis				
Exotropia	7	10	17	270 (61.8)
Esotropia	9	4	13	167 (38.2)
Diplopia				
Yes	8	4	12	160 (36.6)
No	8	10	18	277 (63.4)

The questionnaires were distributed to 451 patients; and a total of 448 questionnaires were returned, with a response rate of 99.3%. Eleven of the questionnaires were not fully completed (missing data ≥30.0%). Thus, 437 (97. 5%) questionnaires were valid for further statistical analysis (mean age, 29.58 ± 11.24 years; range, 18 ~ 64). Table 1 shows that over 60% of the participants were under age 30. Only seventy-one (16.2%) reported an education level of university and above. Approximately 75% of the respondents claimed to have family support. A total of 260 (61.8%) of the patients had exotropic strabismus, and 160 (36.6%) suffered from double vision.

### Self-reported strabismus symptoms

A list of the primary symptoms experienced by the interviewees is shown in Table 2. The most commonly reported symptoms (*n* ≥ 50%) were blurred vision, monocular vision and physical discomfort (i.e., pain, redness, soreness and itch). Other common symptoms included eye fatigue, cannot estimate depth well, increasing deviation size, loss of sight on one eye, sensitive to light, and compensatory body posture. Less than 15% of the patients reported the symptom of limited visual field. Compared to those without diplopia, the patients who suffered diplopia reported more often reported the symptoms of “eye fatigue” (*p* = 0.02) and “cannot estimate depth well” (*p* = 0.02).

**Table 2:** Self-reported symptoms among adult patients with strabismus with and without diplopia

Symptoms as reported	Total (*N* = 30) (%)	Diplopia (n = 12) (%)	Non-diplopia (*n* = 18) (%)	*x* ^*2*^	*P*
Blurred vision	20 (66.7)	8 (66.7)	12 (66.7)	0.00	1.00
Monocular vision	16 (53.3)	7 (58.3)	9 (50.0)	0.20	0.65
Physical discomfort (i.e., pain, redness, sore, and itch)	15 (50.0)	6 (50.0)	9 (50.0)	0.00	1.00
Eye fatigue	10 (43.3)	7 (58.3)	3 (16.7)	5.63	0.02*
Fail to estimate depth well	8 (26.7)	6 (50.0)	2 (11.1)	5.57	0.02*
Increasing deviation size	8 (26.7)	5 (41.7)	3 (16.7)	2.30	0.13
Loss of sight on one eye	7 (23.3)	2 (16.7)	5 (27.8)	0.50	0.48
Sensitive to light	7 (23.3)	3 (25.0)	4 (22.2)	0.03	0.86
Compensatory body posture	6 (20.0)	2 (16.7)	4 (22.2)	0.14	0.71
Limited visual field	4 (13.3)	2 (16.7)	2 (11.1)	0.19	0.66

* *p* < 0.05, two sides

In the survey, the top three symptoms (*n* ≥ 70%) rated by the participants were monocular vision, eye fatigue and physical discomfort. Compared to those without diplopia, the patients who suffered diplopia reported more often reported the symptoms of “blurred vision”, “monocular vision”, “physical discomfort”, “eye fatigue”, “cannot estimate depth well” and “increasing deviation size” (all *p* < 0.05) (Table 3).

**Table 3:** Self-reported symptoms among adult patients with strabismus with and without diplopia

Symptoms as reported	Total (*N* = 437) (%)	Diplopia (*n* = 160) (%)	Non-diplopia (*n* = 277) (%)	*x* ^*2*^	*P*
Blurred vision	282 (64.5)	123 (76.9)	159 (57.4)	15.96	0.00*
Monocular vision	330 (75.5)	132 (82.5)	198 (71.5)	6.08	0.01*
Physical discomfort (i.e., pain, redness, sore, and itch)	311 (71.2)	128 (80.0)	183 (58.8)	8.93	0.00*
Eye fatigue	359 (82.2)	141(88.1)	218 (78.7)	5.52	0.01*
Fail to estimate depth well	267 (61.1)	118 (73.8)	149 (53.8)	16.17	0.00*
Increasing deviation size	286 (65.4)	119 (74.4)	167 (60.3)	8.29	0.00*
Loss of sight on one eye	131 (30.0)	56 (35.0)	75 (27.1)	2.67	0.08
Sensitive to light	–	**–**	**–**	**–**	**–**
Compensatory body posture	205 (46.9)	80 (50.0)	125 (45.1)	0.78	0.37
Limited visual field	213 (48.7)	87 (54.4)	126 (45.5)	2.86	0.08

* *p <* 0.05, two sides

### Impact of strabismus on patients’ HRQoL

Five themes were revealed regarding the impact of strabismus on the patients’ HRQoL: appearance, daily activities, personal development, social interaction, and emotions. The conceptual model was developed according to the Wilson and Cleary model for health-related quality of life [24]. In-depth descriptions for each theme are shown below, with selected quotations from the patients.

#### Appearance

Due to asymmetry in eyes, over two-thirds (76%) of the participants mentioned the negative impact of strabismus on appearance; this impact was mentioned spontaneously by several participants, without being prompted. Participants also reported avoiding looking into a mirror or taking a photo because of feeling inferior about their appearance. This dissatisfaction with appearance reduced the participants’ chance of getting a job or finding a partner; therefore, undergoing cosmetic surgery to correct unsymmetrical eyes was the top reason for young adults with strabismus to seek medical help.

*“The most significant impact for me is appearance. Two eyes are not symmetrical. I cannot even look at myself in a mirror.” (Ms. B, patient without diplopia, 24 yrs, unmarried, unemployed)*

*“The only reason I came here is to correct my eyes. I am not expecting to improve my eyesight anymore.… I never or seldom take a photo because I do not like my appearance, and I do not want to give others a chance to laugh at my eyes when they look at the photo.” (Mr. S, a patient with diplopia, 21 yrs, unmarried, university student)*

*“I wanna become beautiful. I wanna look like a normal person. My parents did not allow me to have surgery at first. But when they realized that my appearance has deeply influenced my chance of getting a job and having a relationship, they finally agreed.” (Ms. DD, a patient without diplopia, 23 yrs, unmarried, unemployed)*

#### Daily activities

Symptoms, such as poor depth perception, eye fatigue, double vision and limited visual field have a substantial impact on patients’ daily activities. Most of the complaints were about having difficulties with activities, such as cooking, doing sports, driving, watching TV, and reading newspapers. Nine interviewees described failing to enjoy hobbies due to double vision. Some participants (30%) complained that double vision and poor eyesight were risk factors for falling and unintentional injuries. Over half of the male participants were upset about that they could not drive. Some were not allowed by law to obtain a driving licence, while others had to quit driving for safety reasons.

*“My two eyes cannot focus on one point, so I often missed the hook that I was trying to hang something on. I thought that I had the right directions, but actually I missed the target….I gave up playing badminton and ping-pong since I cannot locate the ball exactly. It is also a pity that I am not allowed to drive a car.” (Mr. P, a patient with diplopia, 42 yrs, married, forestry officer)*

*“It is very inconvenient and dangerous for me to ride a motorbike. There was a time when I was riding a motorbike; I suddenly saw two cars appearing in front of me. I fell down because I wanted to escape them. But then I found there was actually only one car. I saw two because of my double vision. This kind of thing happened several times.” (Mr. H, a patient with diplopia, 42 yrs, married, technician)*

*“I lost eyesight in my right eye. I have to depend on my left eye. … Since you can only use one eye to see, you feel like that the ground was uneven while you are walking…If a step was not marked, I might miss it and then fall. The biggest potential danger for me is falling.” (Mr. Z, a patient without diplopia, 60 yrs, married, retired)*

*“I am so fond of sewing, knitting and embroidery. Now I have to abandon them due to my strabismus.” (Ms. F, a patient without diplopia, 18 yrs, unmarried, unemployed)*

#### Personal development in education and career

Most participants (76%) discussed the limitations on personal development, in terms of educational and occupational opportunities. Seven interviewees mentioned that they could not choose a major that they would like to study, and half of the participants discussed the experience of not being treated equally when applying for a job position. Seven participants expressed their concerns about their working abilities, including difficulty finding work, reduced working hours and slower work speeds than people with normal eyesight. Three participants regretted that they had to abandon job promotions because of the low self-efficacy associated with strabismus.

*“My life would be different if my eyes were all right. When I was young, I was fond of and good at physical sports. My dream was becoming a physical education teacher. But I failed to enter in a sport college because I lost my eyesight in my right eye.” (Mr. E, a patient without diplopia, 32 yrs, unmarried, chef)*

*“I had a strong willingness to study. I deeply understood that studying would change my life. But I had to drop out of school since I failed to see the words written on a blackboard, even sitting in the first row. …I often quit opportunities of continuous education. It is troublesome to travel around with my poor eyesight. Besides, I know that more learning is no help to my job, since my eyes have already limited my career development.” (Mr. N, a patient with diplopia, 26 yrs, married, staff).*

*“I can tell you that no one would like to employ me as a waitress. I can only be offered a job as dishwasher, because no guest will notice my eyes when I hide in a corner to do washing. … Besides, I was often misunderstood by employers that I had poor eyesight; but actually, my eyesight is quite good.” (Mrs. Y, a patient without diplopia, 41 yrs, married, unemployed)*

*“The main reason I came here for a surgery was that I wanna update my driving licence, from driving a shuttle to driving a bus. But my application was denied because of my strabismus. I need to correct my eyes before I re-submit an application. …If my driving license cannot get updated after the surgery, I will probably run a small business so I can avoid the stigma from other employers.” (Mrs. L, a patient without diplopia, 39 yrs, divorced, shuttle driver)*

#### Social interaction

More than 20 participants showed a strong willingness to get involve in social activities, and they regarded the interaction with family and friends as a main source of happiness and life satisfaction; however, having strabismus had caused the interviewees to reduce or even avoid having social lives because of the social discrimination associated with strabismus, which normally presented in three manners: teasing and mocking, being misunderstood, and being hidden. First, it was common for strabismus patients to be mocked and teased by classmates, colleagues, teachers, and strangers. They were given awful nicknames due to strabismus. Seven participants experienced an updated conflict of quarrelling and fighting because they failed to tolerate tease or mock. Second, over one-third of the participants (36.7%) complained that their asymmetrical eyes were misunderstood as being unfriendly or impolite when they failed to control their eyes in the same direction. They tried to look straight at the people they were talking to, but their eye balls just went moved upward or to the other side. Third, five interviewees mentioned that they were hidden by others to avoid interpersonal activities with the strabismus patients. They assumed that it was because that strabismus was regarded as a kind of physical disability and that their appearance frightened others.

*“I think I am a lucky person. I am divorced and I am disabled on eyes, but everyone I encountered treats me well. I do not know whether this is because of my eyes. They sympathize with me, so they treat me well. Anyway, I still feel lucky. But I reduce the time of going out with them because I did not want to cause them too much trouble. I am a disabled person. I am different from them.” (Mrs. L)*

*“Other women will misunderstand and suspect me. When I talked to a woman while her husband by her side, she thought I was looking at her husband in a sexual manner. Actually, I was not. I was looking at her! But my eyes were squinting, and it looked like straight to her husband.” (Mrs. Y, a patient without diplopia, 41 yrs, married, unemployed)*

*“If I wanted to see things clearly, then my eyes were squint; if I controlled my eyes in orthotropic position, I failed to see things clearly. I did not want my friends to know that I had strabismus, so I preferred to control my eyes in an orthotropic position rather than seeing things clearly…. Then trouble comes. When a friend was walking and waving toward me, I could not see him. He thought that I was looking down upon him. But you know, I was not. I just could not see him when I tried to keep my eyes in orthotropic position.” (Mr. H, a patient without diplopia, 61 yrs, married, retired)*

*“I felt so lonely because I could not find someone look like me. I did not know where I should go to talk about my unhappiness associated with my eyes. I went to internet and blogs. I joined a “disabled group”, trying to find someone who could understand me.” (Mr. E)*

#### Emotions

All participants discussed the negative emotions resulting from strabismus. The emotions that most commonly complained were anxiety, sadness, and inferiority. Other emotional impacts were reported including angry, embarrassment and irritability.

The anxious feelings came from four aspects: (1) worrying that the strabismus was getting worse, such as increasing deviation size and decreasing eyesight, (2) worrying that corrective surgery might cause blindness; (3) worrying about family or work affairs while taking surgery, (4) worrying about the negative impacts of strabismus on life (e.g., unemployment, safety risks, find a partner). The feelings of sadness and self-inferiority resulted from the disability of eyes and the tease associated with strabismus from others.

*“My strabismus is getting worse and worse, and I am worried about losing eyesight. But speaking of the (corrective) surgery, I am also afraid of infection after the surgery. I kept worrying since eyes are important organs. I have no idea of what my life would be like if I lose my eyes.” (Ms. C, a patient without diplopia, 21 yrs, unmarried, student)*

*“Currently the only thing I am worried about is that I cannot find a girl to get married. I mean, because of my eyes, I do not deserve a nice wife.” (Mr. D, a patient without diplopia, 25 yrs, unmarried, chef)*

*“People will look down upon you because of my eyes. When people around you keep saying that you are “monster” “freak”, when they kept laughing at you, you would feel inferiority in the end.” (Mr. M, a patient without diplopia, 43 yrs, married, self-employed)*

### Correlation of the themes that emerged in the interview

The overall score of symptom items showed significantly adequate correlation with the scores of all other themes (*r* = 0.336~ 0.567, *p* < 0.05), except for the theme of “appearance” (*r* = 0.205, *p* < 0.05) (Table 4). The score of “emotions” theme had significantly moderate to strong correlation with the scores of other four themes, with coefficients ranging from 0.340 to 0.784 (*p* < 0.01) (Table 4). The theme of “social interaction” showed a statistically evident relationship with the theme of “appearance” (*r* = 0.570, *p* < 0.05), “daily activities” (*r* = 0.453, *p* < 0.05) and “personal development” (*r* = 0.587, *p* < 0.05) (Table 4). A significantly strong correlation was found between the themes of “daily activities” and “personal development” (*r* = 0.599, *p* < 0.05) (Table 4).

**Table 4:** Correlation between scores of each subscale and the total score

Items^a^	Symptoms	Appearance	Daily activities	Personal development	Social interaction
Symptoms	1				
Appearance	0.205**	**1**			
Daily activities	0.567**	0.340**	**1**		
Personal development	0.362**	0.366**	0.599**	**1**	
Social interaction	0.336**	0.570**	0.453**	0.587**	**1**
Emotions	0.340**	0.630**	0.525**	0.571**	0.784**

Pearson coefficient. ** *p <*0.01, two-sides

^a^Negative impacts of symptoms on appearance, daily activities, personal development, social interaction, and emotions

### Differences in symptoms burden between patients with and without diplopia

Adult patients with diplopia reported significantly more negative symptoms compared to those without diplopia. The qualitative data revealed that strabismus patients with diplopia complained more than those without diplopia about eye fatigue (*χ*^*2*^ = 5.63, *p* = 0.02) and the failure to estimate depth well (*χ*^*2*^ = 5.57, *p* = 0.02) (Table 2). As shown in Table 3, strabismus patients with diplopia reported significantly higher levels of symptom burden than those without diplopia, in terms of blurred vision (*χ*^*2*^ = 15.96, *p* = 0.00), monocular vision (*χ*^*2*^ = 6.08, *P* = 0.01), physical discomfort (*χ*^*2*^ = 8.93, *p* = 0.00), eye fatigue (*χ*^*2*^ = 5.52, *p* = 0.01), failure to estimate depth well (*χ*^*2*^ = 16.17, *p* = 0.00) and increased deviation size (*χ*^*2*^ = 8.29, *p* = 0.00).

## Discussion

**Fig. 1:**
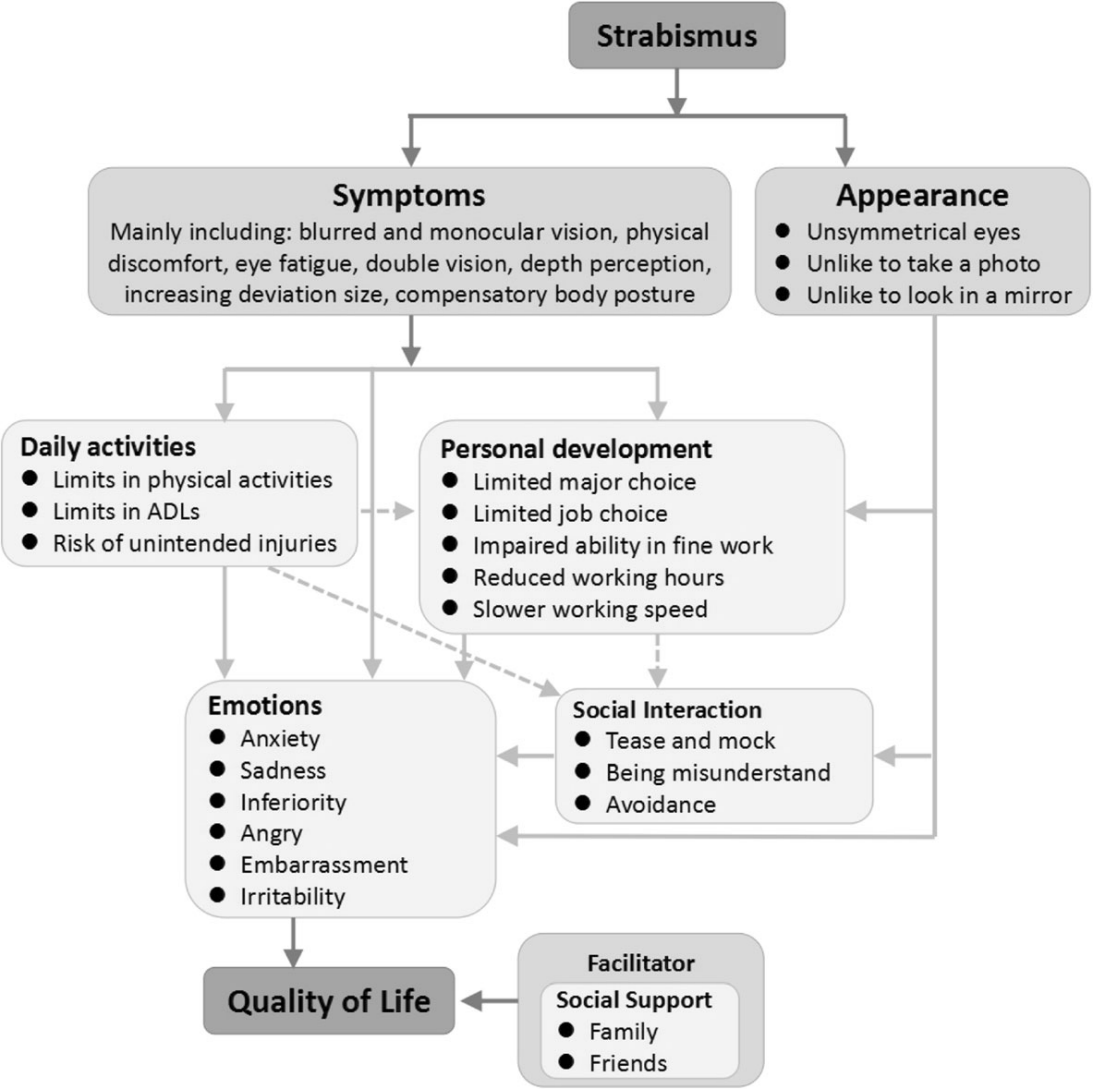
Conceptual model of health-related quality of life for strabismus

The study examined the impact of having strabismus on patients’ QoL through qualitative interviews and cross-sectional survey, and accordingly, a conceptual model was developed from the patients’ perspectives (Fig. 1). The model lists a series of strabismus symptoms complained frequently by the patients, including blurred and monocular vision, physical discomfort, eye fatigue, double vision, depth perception, increasing deviation size, compensatory body posture. Symptoms burden and appearance are the two original reasons for the decrease of QoL, and they are also the triggers for strabismus patients to visit clinics and undergo surgery. Although these symptoms are not directly responsible for the declined strabismus-related QoL, the symptoms do place a wide-ranging detrimental impact on the patients’ daily activities, personal development, emotions and social interaction, which consequently contributed to the declined QoL. This finding implies that the QoL of strabismus patients could be greatly improved if the problems regarding the symptoms and appearance could be solved, and these improvements could positively impact their daily activities, personal development, emotions and social interaction.

The management of symptoms should be considered when treating adult strabismus patients. The participants in the study reported a high level of disturbing symptoms related to strabismus. The symptoms burden of blurred vision, monocular vision, physical discomfort in eyes and eye fatigue were commonly complained. As the conceptual model shows, although the symptoms burden was not directly responsible for the declined strabismus-related QoL, the symptoms did negatively impact the patients’ daily activities, personal development, emotions and social interaction, which consequently contributed to the declined QoL. The findings were consistent with those of previous studies. Simonsz and colleagues [3] have collected complaints about how amblyopia and strabismus affected daily life from outpatients and then summarized into five main domains: “fear of losing the better eye”, “distance estimation”, “visual disorientation”, “diplopia” and “problems with social contact and cosmetic problems”. In another qualitative study, the most frequent complaints mentioned by adults with strabismus were extracted from an analysis of 30 interviews [23], and it was found that negative feelings, general disability, difficulties with driving, interpersonal relationships and problems with eye contact were the primary issues.

The apparent impacts of strabismus symptoms on QoL may also explain why QoL could be significantly improved after a strabismus surgery. According to our conceptual model, it was reasonable to assume that physical and psychosocial functions and overall QoL could be greatly improved after the relief of strabismus symptoms. This hypothesis has been proven by a series of previous studies that strabismus surgery could diminish or at least alleviate strabismus symptoms, and further improved patients’ psychological and functional outcomes [17, 19, 22, 25, 26]. For example, in Holmes and et al.’s study [17], the overall scores of adult strabismus questionnaire (AS-20) were significantly improved from 6 weeks to 1 year following strabismus surgery among adult patients, as were the psychological and function subscale scores. The long-lasting, positive impacts on the HRQoL were particularly significant when the patients remain successfully aligned and diplopia-free after surgery. As the authors concluded, “these data provide further evidence of the comprehensive HRQoL benefits of strabismus surgery”. In another study by Nelson et al. [27], they examined psychosocial concerns and satisfaction before and after corrective surgery in 128 teenagers and adults. Following surgery, most participants reported their satisfaction with the ocular realignment and the improvements in their self-esteem, abilities to meet new people and interpersonal relationships. In addition to the improvements in QoL and satisfaction after strabismus surgery, the levels of anxiety and depression have been significantly decreased [26]. Although strabismus surgery has been regarded as the most effective method of improving patient QoL, 84% of the patients still believed that their eyes were still not aligned after the surgery [28], highlighting a possible persistent effect on QoL.

An increasing number of studies have found a distinction between patients with and without diplopia, although a number of common concerns were reported across the two groups. It was revealed that adult patients with diplopia predominantly reported physical disabilities and difficulties in vision-related functions, such as fine work and driving, while those without difficulties showed more concerns about appearance and psychosocial functioning [29]. According to previous literature and our qualitative findings, the possible reason we assumed for this difference was that double vision contributed greatly to the high risk of falling and unintended injuries through impaired eye functions. For the patients with diplopia, as their basic demands of individual safety were threatened, it was no wonder that psychosocial difficulties became less of a concern. The significant differences between the patients with and without diplopia were also examined in our survey. As shown in Table 3, patients with diplopia were more likely to be disturbed by strabismus symptoms in contrast to those without diplopia. It is necessary to note that corrective surgery could not always successfully eliminate diplopia, particularly if strabismus develops after the age of visual maturity. In a prospective study, only 57% of patients with diplopia were found to have had no diplopia post operation [30]. Therefore, suggestions to ophthalmologists and nurses are that more attention should be placed on strabismus patients with diplopia both before and after surgery, and more clinical research are needed to explore effective interventions to treat diplopia. Currently several studies have focused on the development of instrument to better quantify the diplopia and in the exploration of more effective treatments [30–33].

As shown in the conceptual model (Fig. 1), symptom burden and appearance were the two original reasons for the decrease of QoL, and they were also the triggers for strabismus patients to visit clinics and undergo surgery [34]. The eyes were regarded as being particularly important to facial appearance and perceived attractiveness. Unsurprisingly, ocular misalignment exerted considerable impacts on QoL. Appearance cannot decrease the QoL directly, but it can affect QoL by influencing a person’s self-appraisal, emotions and social contact. Particularly in Chinese culture, the face is closely associated with a person’s social status and feelings of embarrassment, dignity and disgrace. There was even a traditional saying, “losing face”, which was used to describe the status of feeling shame, awkward and indignity. Realignment surgery could improve appearance greatly. Therefore, it was generally regarded as cosmetic, and adult strabismus surgery was not covered by Chinese health insurance. In our interview, this issue was spontaneously brought up by the participants. As strabismus patients usually have lower levels of education and income, compared to unaffected adults [11], the surgery fee was an economic burden to them, particularly for those who had to undergo surgery several times. The primary obstacle for their delayed treatment to adulthood was their ignorance of the disease. Most patients thought that their appearance and strabismus were a curse and could not be cured. In this sense, health education should be provided to the public, particularly to parents, to raise their awareness about strabismus, its long-lasting negative impacts and its early interventions.

## Conclusions

To the best of our knowledge, this study is the first to examine quality of life among Chinese strabismus patients by using a mixed-method approach, and based on the results, we proposed a conceptual model. The conceptual model was expected to provide readers a framework about the various concepts that were associated with the strabismus-specific QoL and the links between the concepts. Moreover, it could enlighten the potential targets to develop interventions to improve QoL.

As shown in the study, symptoms burden and poor appearance were the two primary reasons for the declined QoL in patients with strabismus, and these two factors were also the triggers for patients undergoing surgery. Therefore, eliminating strabismus-related symptoms should be addressed first when improving QoL. In addition, more consideration should be given to patients with diplopia.

## Abbreviations

AS-20: Adult Strabismus Questionnaire
HRQoL: health-related quality of life
